# Programmed cycle-induced endometrial perturbations do not independently influence angiogenic imbalance or hypertensive disorders in pregnancy

**DOI:** 10.1172/jci.insight.202443

**Published:** 2026-06-23

**Authors:** David Huang, Emily Flynn, Brittany Davidson, Juan C. Irwin, Mohammad Naser, Ana Laura Almonte, Jennifer Qin, Yue Song, Fleurdeliza B. Rabara, Rebecca Wong, Lydia B. Zablotska, Mitchell P. Rosen, Torsten Wittmann, Gabriela K. Fragiadakis, Alexis J. Combes, Marina Sirota, Marcelle I. Cedars, Linda C. Giudice

**Affiliations:** 1 Division of Reproductive Endocrinology & Infertility, Department of Obstetrics, Gynecology & Reproductive Sciences,; 2 Center for Reproductive Sciences,; 3 CoLabs,; 4 Bakar Computational Health Sciences Institute,; 5 Biomedical Informatics Program,; 6 Department of Obstetrics, Gynecology & Reproductive Sciences,; 7 Department of Epidemiology and Biostatistics,; 8 Division of Rheumatology, Department of Medicine, and; 9 Department of Pathology, Division of Gastroenterology, Department of Medicine, Bakar ImmunoX Initiative, UCSF, San Francisco, California, USA.

**Keywords:** Clinical Research, Reproductive biology, Fertility, Obstetrics/gynecology, Translation

## Abstract

**BACKGROUND:**

In vitro fertilization (IVF) culminates in embryo transfer into a hormonally primed endometrium, often via a programmed cycle (PC) regimen postulated to influence hypertensive disorders of pregnancy (HDP) risk. We thus generated a single-cell atlas of PC endometrium to define cell type–specific differences relative to natural cycle (NC) endometrium and evaluated whether PC-associated modulation of the window of implantation (WOI) endometrium influences angiogenic balance in pregnancy.

**METHODS:**

snRNA-seq of prospectively collected PC and NC WOI endometrium. An independent prospective cohort of 548 singleton pregnancies was separately analyzed for maternal serum angiogenic markers (soluble fms-like tyrosine kinase-1; placental growth factor) and HDP incidence in PC- versus NC-conceived pregnancies, adjusting for clinical confounders and IVF use.

**RESULTS:**

Prominent transcriptomic differences were observed between PC (*n* = 7; 48,843 nuclei) and NC (*n* = 9; 44,230 nuclei) WOI endometrium, particularly in glandular epithelium (682 up- and 979 downregulated genes; *P*_adj_ < 0.05) and stromal fibroblasts (108 up- and 168 downregulated). PC endometrium showed reduced uterine natural killer cell abundance, potentially from *CXCL14* downregulation. Functional enrichment revealed downregulation of embryo implantation, angiogenesis, and extracellular matrix remodeling pathways in PC. Altered cell-cell signaling in decidualization, angiogenesis and inflammatory response were also observed. Despite these WOI perturbations, PC-conceived pregnancies were not associated with early gestational angiogenic imbalance or increased HDP risk.

**CONCLUSION:**

PC endometrial preparation induced distinct cellular and signaling alterations in the WOI but was not associated with subsequent development of angiogenic imbalance or HDP, thereby underscoring the resilience and adaptability of the early maternal-fetal interface.

**TRIAL REGISTRATION:**

ClinicalTrials.gov NCT03799107.

**FUNDING:**

ABOG/AAOGF; NICHD-R01-HD084380; NCTRI-P50-HD055764; NIAMS-P30-AR070155.

## Abstract 2

Programmed-cycle endometrium in IVF showed cell type-specific perturbations, but without subsequent angiogenic imbalance or increased hypertensive disorder in pregnancy, highlighting adaptability of the maternal-fetal interface.

## Introduction

Assisted reproductive technology (ART) is increasingly utilized worldwide for both fertility treatment and elective fertility preservation (1, 2). Over the past decade, ART laboratory techniques have also progressed considerably. With evolving ART practices and expanded clinical indications, the number of embryo cryopreservation cycles — and consequently, frozen embryo transfers (FETs) — has steadily increased (1, 3). When cryopreserved embryos are thawed for transfer, endometrial preparation is required to support implantation. There are 2 main methods for endometrial preparation in FET: the natural cycle (NC) and the programmed cycle (PC). NC endometrial preparation relies on the endogenous hypothalamic/pituitary/ovarian (HPO) axis for follicular recruitment and progesterone production, while PC endometrial preparation mimics the hormonal sequence of NC exclusively with exogenous hormone replacement (Supplemental Table 2; supplemental material available online with this article; https://doi.org/10.1172/jci.insight.202443DS1). Exogenous hormones in PC FET suppress the endogenous HPO signaling, preventing follicular development and ovulation, leading to the absence of a corpus luteum and its associated products during the window of implantation (WOI).

While both NC and PC endometrial preparation methods are sufficient for implantation, debate continues over which method is superior, both for pregnancy rates as well as obstetric outcomes (4–6). FET success rates with euploid/good morphology blastocysts have plateaued at 50%–65% per transfer, suggesting a potential endometrial contribution that remains elusive at the clinical level (7). Therefore, recent studies have also emerged to focus on the human WOI endometrium using single-cell sequencing technologies, improving resolution and understanding of the various cell types’ roles in establishing a receptive environment for implantation (8, 9). However, studies have not systematically evaluated global transcriptomic differences at a single-cell level between NC and PC endometrial environments.

Furthermore, some studies have also reported an increased risk of hypertensive disorders of pregnancy (HDP) following PC FET compared with NC FET (10, 11), while others have not (12, 13). One possible mechanism is the absence of a corpus luteum and, consequently, reduced circulating relaxin in early pregnancy following PC FET (14). Relaxin, a hormone primarily produced by the corpus luteum, has vasodilatory effects, enhances arterial compliance (15, 16), and promotes in vitro endometrial stromal decidualization (17, 18). Despite biological plausibility, meta-analyses focusing on HDP risk associated with PC FET have highlighted significant methodological limitations, including study design heterogeneity, quality of evidence, and critically, the lack of an appropriate comparison group to establish reference HDP risk in patients with infertility who conceived without ART (10, 19). Recent investigations showed that many associations between ART and obstetric complications were attenuated or not significant after adjusting for infertility diagnoses or fertility status (20–22). To better examine the specific risks associated with PC FET, prospective clinical data are needed to assess HDP risk uniquely attributed to different endometrial preparation methods while accounting for underlying infertility, IVF use, and other known risk factors. Recent clinical advances also included development of soluble fms-like tyrosine kinase-1 (sFlt-1) and placental growth factor (PlGF). These 2 markers are circulating serum analytes of maternal endothelial dysfunction, and sFlt-1/PlGF ratios have demonstrated clinical predictive value for HDP and preeclampsia (23–25).

To investigate how NC and PC differs in their WOI environment and resultant clinical implications, we sought to characterize fundamental differences between NC and PC WOI endometrium at single-cell resolution and to assess whether changes in WOI signatures correlate with subsequent angiogenic imbalance in pregnancy using an independent clinical cohort with rigorously designed comparator groups. We hypothesized that PC endometrial preparation alters key reproductive pathways during the WOI and that these perturbations may predispose to a suboptimal environment for early placentation and spiral artery modeling, thereby manifesting as early angiogenic imbalance and/or an increased HDP risk. To clinically correlate these molecular differences with the hypothesized increased HDP risk after PC FET, we compared early pregnancy maternal sFlt-1 and PlGF measured between 6 and 20 weeks’ gestation, as well as physician-adjudicated HDP diagnoses, in an independent prospective cohort of pregnancies conceived under NC or PC conditions, adjusting for underlying infertility, relevant clinical confounders, and ART use.

## Results

### Global differences in transcriptomic signature and cellular topography in PC endometrium.

To rigorously isolate the effects of PC endometrial preparation on the global WOI endometrial environment, we first compared PC (*n* = 7) to NC (*n* = 9) WOI endometrium using single-nucleus transcriptomic analysis (snRNA-seq). The samples were processed in a single batch using tissue from patients with unexplained infertility and normal endometrial cavity evaluation. Additional relevant clinical data of these 16 patients and their hormonal exposures are summarized (Supplemental Tables 1 and 2).

**Figure 1:**
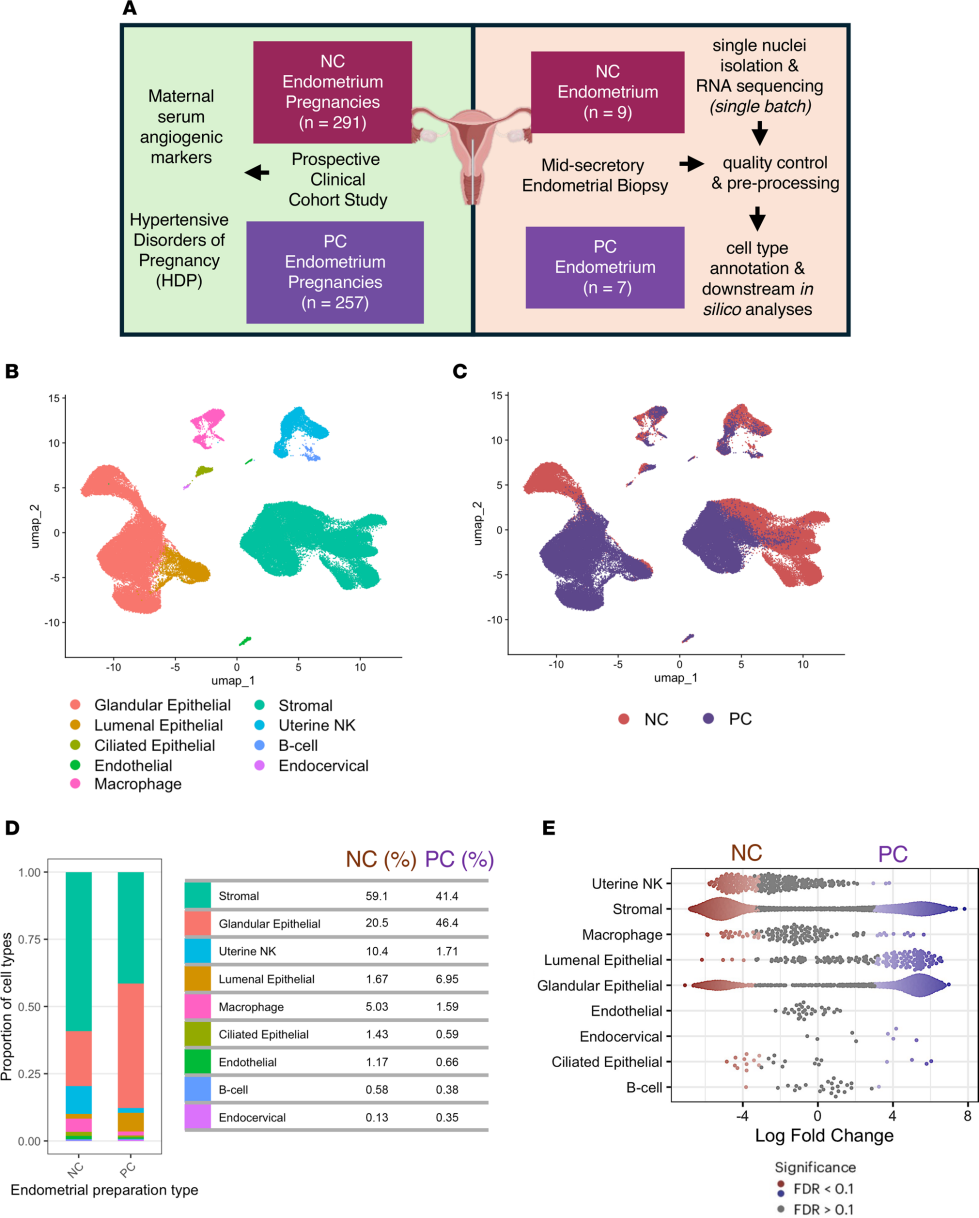
snRNA-seq of WOI human endometrium in NC and PC settings. (**A**) Study methodology combining single-nucleus transcriptomics (left) and a prospective clinical cohort study (right) to examine differences between NC and PC endometrium at both molecular and clinical levels. (**B**) Uniform manifold approximation and projection (UMAP) visualization of snRNA data from a total of 16 individuals and 93,064 nuclei after quality control measures and cell type annotation. (**C**) UMAP visualization of WOI endometrium transcriptomes by endometrial preparation method (NC and PC). (**D**) Bar plot depicting the cellular compositions of NC and PC endometrium. (**E**) Beeswarm plot showing distribution of log-fold change (FC) of various cell type neighborhoods by endometrial preparation method. Differentially abundant neighborhoods with |logFC| > 2.5 and Spatial FDR < 0.1 are colored.

Single nuclei were extracted from WOI endometrial biopsy samples and sequenced using the 10x Genomics platform (Figure 1A). After quality control, a total of 93,064 nuclei were retained for final analyses: 44,230 nuclei (47.5%) from NC endometrium and 48,834 nuclei (52.5%) from PC endometrium samples. Five major cell types were identified by cell lineage marker genes (Supplemental Figure 1): epithelial (glandular, luminal, and ciliated subtypes; total *n* = 36,762), stromal fibroblasts (*n* = 46,354), immune (macrophage, uterine natural killer, and B cell; total *n* = 8,878), endothelial (*n* = 840), and endocervical (*n* = 230) (Figure 1B). We were not able to confidently map T cells in this dataset, which could be secondary to the low number of T cells in the secretory phase (26), and/or lower sensitivity for this cell type intrinsic to snRNA processing (27). Global transcriptomic differences were observed between PC and NC WOI endometrium using UMAP dimensionality reduction (Figure 1C). The degree of transcriptomic changes associated with PC preparation was not uniformly observed across all endometrial cell types. Transcriptomic signatures were markedly altered in glandular epithelial and stromal fibroblast cells, with clear clustering on UMAP by endometrial preparation method in these 2 populations (Figure 1C). Proportion of variance component analysis estimated that 30.0% of variance in principal component (PC) expression space among glandular epithelium is explained by endometrial preparation method, and 16.0% in stromal fibroblasts. Ciliated epithelium also showed distinct transcriptomic signatures in PC compared with NC, with 11.7% of variance in PC-expression space explained by endometrial preparation method (Supplemental Figure 2A). Other cell types showed less discernible clustering between NC and PC, all with less than 5.0% of variance in PC-expression space attributed to endometrial preparation method (Supplemental Figure 2, B–F).

We next examined the cellular composition of WOI NC and PC endometrium. The relative proportions of identified cell types by endometrial preparation method are shown in Figure 1D. The epithelial/stromal ratio was higher in PC (ratio: 1.30) compared with NC (ratio: 0.40) endometrium, potentially in the setting of supraphysiologic estradiol exposure, which is similar to that observed in endometrium following ovarian hyperstimulation (28). Strikingly, the percentage of uterine NK cells was significantly lower in PC endometrium (1.71%) compared with NC endometrium (10.4%). To examine differential abundance at increased granularity, we applied miloR to the data to identify and assess patterns in cellular neighborhoods by cell type in NC and PC endometrium (Figure 1E) (29). We identified 1,507 and 1,869 neighborhoods that were significantly enriched (spatial FDR <0.1) in PC and NC, respectively. Similar to the coarse cell type results, only 3 uterine NK cell neighborhoods were significantly enriched in PC endometrium, compared with 99 uterine NK cell neighborhoods enriched in NC endometrium (binomial test *P* < 0.001). Concordant with the observation of a higher epithelial/stromal ratio in PC endometrium, 964 glandular epithelial neighborhoods were significantly enriched in PC endometrium, compared with 364 in NC (*P* < 0.001). Similarly, 123 and 2 luminal epithelial neighborhoods were significantly enriched in PC and NC endometrium, respectively (*P* < 0.001). Neighborhood enrichment patterns in other cell types were not significantly different by endometrial preparation method (all *P* > 0.05). These findings suggest that PC endometrium exhibits substantial differences in cellular composition from NC endometrium.

### Differentially regulated genes and biological pathways in PC endometrium.

**Figure 2:**
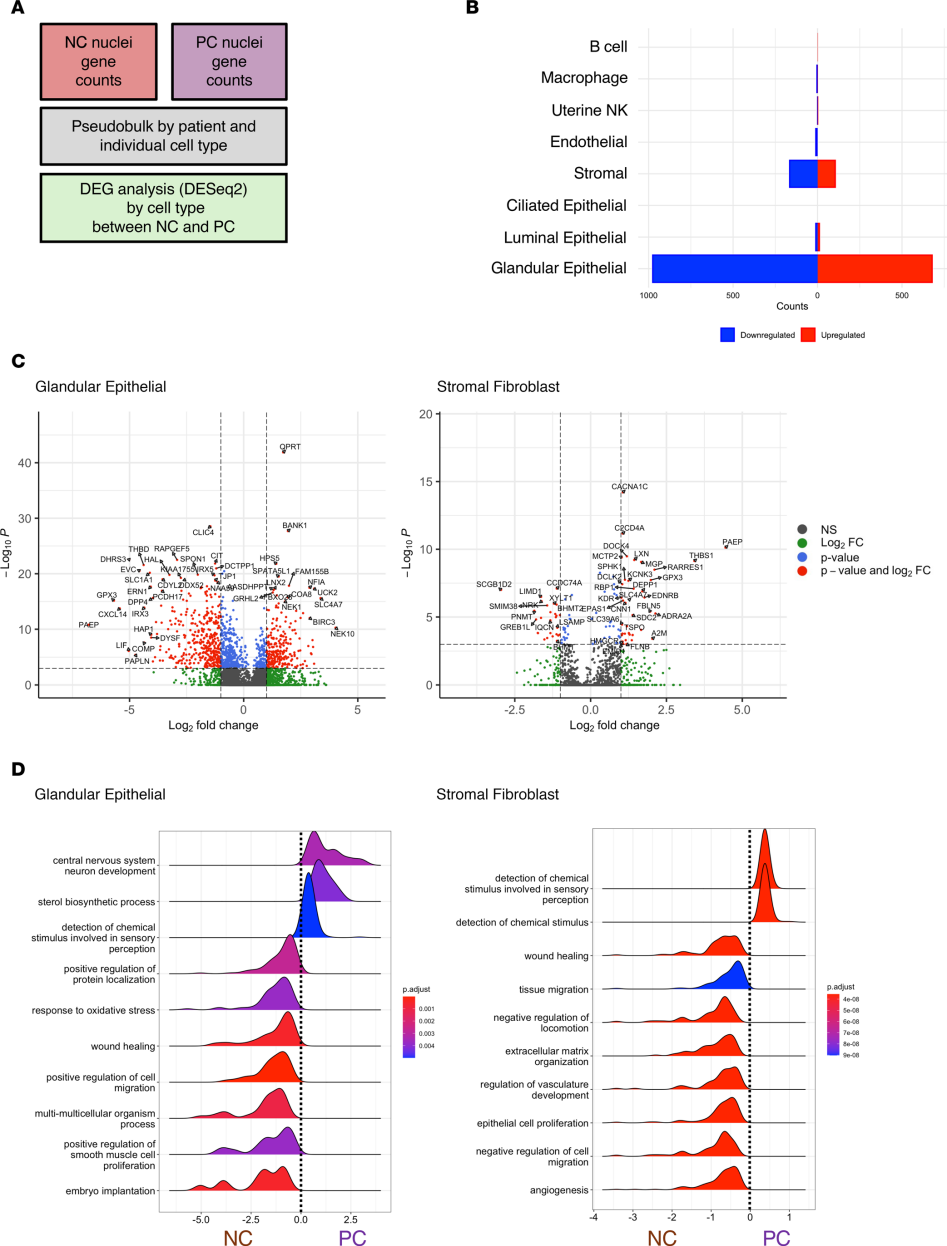
Transcriptomic differences and differentially regulated pathways between natural cycle and programmed cycle window of implantation endometrium. (**A**) Study design to identify differentially expressed genes (DEGs) in programmed cycle (PC) compared with natural cycle (NC) WOI endometrium. (**B**) Number of significant DEGs (|Log_2_FC| > 0.1 and *P*_adj_ < 0.05, DESeq2) by cell type comparing PC to NC endometrium. (**C**) Volcano plots depicting the distribution of DEGs by Log_2_FC value (comparing PC to NC) and –Log_10_ *P*_adj_ (DESeq2) in Glandular Epithelial and Stromal Fibroblast cell types. The most significant DEGs are depicted in red using the dashed lines, which indicate |Log_2_FC| > 1.0 and *P*_adj_ < 0.001 (DESeq2). (**D**) Ridgeline plot demonstrating differentially regulated biological pathways from gene set enrichment analysis using cell type–specific DEGs in glandular epithelial and stromal fibroblasts (*P*_adj_ values from gene set enrichment analysis).

We identified differentially expressed genes (DEGs) by cell type using DESeq2 after pseudobulking to limit false-positive discovery and identify shared per patient effect (Figure 2A). The number of significant DEGs (up- or downregulated in PC endometrium) are shown in Figure 2B. The highest number of significant DEGs (*P*_adj_ < 0.05) were observed in glandular epithelial (682 upregulated; 979 downregulated) and stromal fibroblast populations (108 upregulated; 168 downregulated). In glandular epithelium (Figure 2C), several genes were notably downregulated in PC: *LIF*, *PAEP*, *GPX3*, *COMP*, and *DPP4* (log_2_FC < –1.0, *P*_adj_ < 0.001). These genes were reported to participate in implantation and endometrial decidualization and thought to reflect endometrial receptivity (30–33). *PAEP* and *GPX3* were also significantly downregulated in PC stromal fibroblasts compared with those from NC endometrium (Figure 2C). *THBS1*, *RARRES1*, and *DEPP-1*, which are also genes with proposed roles in decidualization and implantation (32, 34, 35), were also significantly downregulated in PC stromal fibroblasts. These observations suggest that the PC endometrial preparation method may exert cell type–specific effects on postulated key regulators of endometrial receptivity and decidualization, which may influence the early maternal-fetal interface environment.

Given the ongoing debate on optimal endometrial preparation method for fertility treatment, we also examined the relative expressions of markers from a gene panel identified through a meta-analysis (34 epithelial- and 22 stromal-specific markers), whose upregulation is thought to represent the meta-signature of human endometrial receptivity (30). We found that many receptivity markers had differential expression between NC and PC. Eighteen epithelial receptivity markers had significantly lower expression (log_2_FC < –1.0, *P*_adj_ < 0.05) in PC endometrium in our dataset (Supplemental Figure 3). In stromal fibroblasts, 3 markers (*GPX3*, *PAEP*, and *GBP2*) also had significantly lower expression in PC. No receptivity markers in this panel were significantly upregulated in a PC endometrium.

Furthermore, *CXCL14* — a highly conserved homeostatic chemokine — was also significantly downregulated in PC endometrium. *CXCL14* has been shown to modulate chemotaxis, as well as differentiation and activation of various immune cell types (36). *CXCL14*, critically regulated by progesterone, has been shown to exert a chemoattractive effect on uterine NK cells in the endometrium (37). The significantly lower expression of *CXCL14* may explain our finding that uterine NK cells were markedly less abundant in PC endometrium (Figure 1E).

To contextualize the DEGs between PC and NC endometrium, gene set enrichment analyses (GSEA) were performed to identify differentially regulated biological pathways in the setting of exogenous hormone administration and the absence of a corpus luteum (Figure 2D). A total of 156 pathways in glandular epithelium and 615 pathways in stromal fibroblasts were significantly enriched (*P*_adj_ < 0.05) by endometrial preparation method. Gene ontology pathways that were significantly downregulated in PC glandular epithelium were related to protein localization, response to oxidative stress, wound healing, cell migration, and smooth muscle cell proliferation. Concordant with the observed DEGs, embryo implantation also emerged as downregulated in PC from functional enrichment analysis. In PC glandular epithelium, we identified upregulated pathways related to neuron development, sterol biosynthesis, and detection of chemical stimulus. Similarly, detection of chemical stimulus involved in sensory perception was also identified using the upregulated DEGs in PC stromal fibroblasts. The downregulated DEGs in PC stromal fibroblasts heavily mapped to biological pathways related to vascular development, angiogenesis, and extracellular matrix organization, which may have implications for angiogenic balance and remodeling of the endometrium in preparation for implantation and placentation. Negative regulation of cell migration was also seen in functional enrichment analysis from the downregulated DEGs in PC stromal fibroblasts.

### Cell-cell interactome of NC and PC endometrium in the WOI.

**Figure 3:**
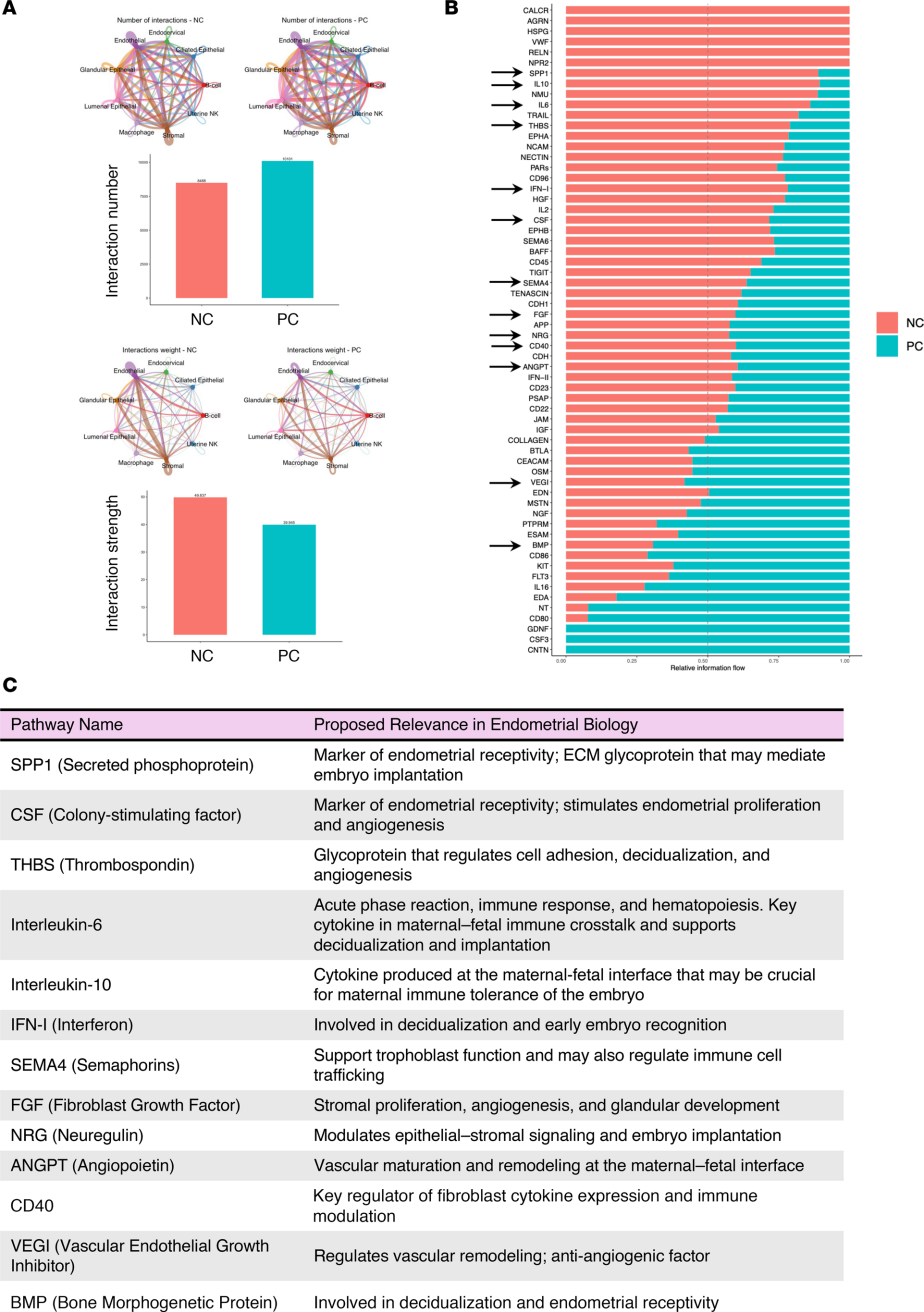
Cell-cell interactome in human window of implantation endometrium in natural cycle and programmed cycle. (**A**) Interaction net count and weight plots in natural cycle (NC) and programmed cycle (PC) window of implantation (WOI) endometrium. (**B**) Relative information flow analysis of biological pathways that demonstrated significant differential enrichment (paired Wilcoxon test *P* < 0.05) between NC and PC WOI endometrium. (**C**) Summary table describing the relevant function of significantly enriched pathways between NC and PC endometrium in reproductive biology

Given the substantial differences in the transcriptomic landscape between NC and PC WOI endometrium, we used CellChat to analyze cell-cell communication patterns of various biological pathways using differentially expressed ligand-receptor pairs (38). Global communication patterns showed that the number of cell-cell interactions are preserved in PC compared with NC endometrium. While there was a higher absolute number of interactions in PC endometrium, the interaction strength appeared higher in NC endometrium (Figure 3A).

To better understand PC-associated changes in the endometrial microenvironment, a bar plot was first used to visualize the relative information flow of signaling pathways that demonstrated significant differences (*P* < 0.05) in relative strength in NC versus PC (Figure 3B). We observed a significant shift in signaling dynamics of 62 pathways between the 2 endometrial preparation methods: 41 showed greater relative information flow in NC, and 21 showed greater relative information flow in PC. A comparative global heatmap of overall signaling patterns in NC and PC WOI endometrium by cell type is also provided (Supplemental Figure 4).

Several of these signaling pathways have been proposed with important functions in reproduction (Figure 3B). For example, signaling pathways involved in endometrial decidualization and stromal homeostasis were more prominent in NC, such as SPP1, THBS, and FGF. Pathways that may have important roles in embryo implantation (SPP1, CSF, SEMA4, and NRG) were also significantly enriched in NC endometrium. Inflammatory modulation (IL-6, IL-10, CD40, and IFN-I) and vascular remodeling (FGF, ANGPT) pathways at the maternal-fetal interface also demonstrated greater signaling activity in NC. On the other hand, pathway involving VEGI — an antiangiogenic factor — was more prominent in PC. The BMP pathway — which has been characterized as an important pathway in endometrial decidualization — was also significantly enriched in PC endometrium. A summary table describing the relevant function of these pathways in endometrial biology is provided in Figure 3C (34, 39–46).

**Figure 4:**
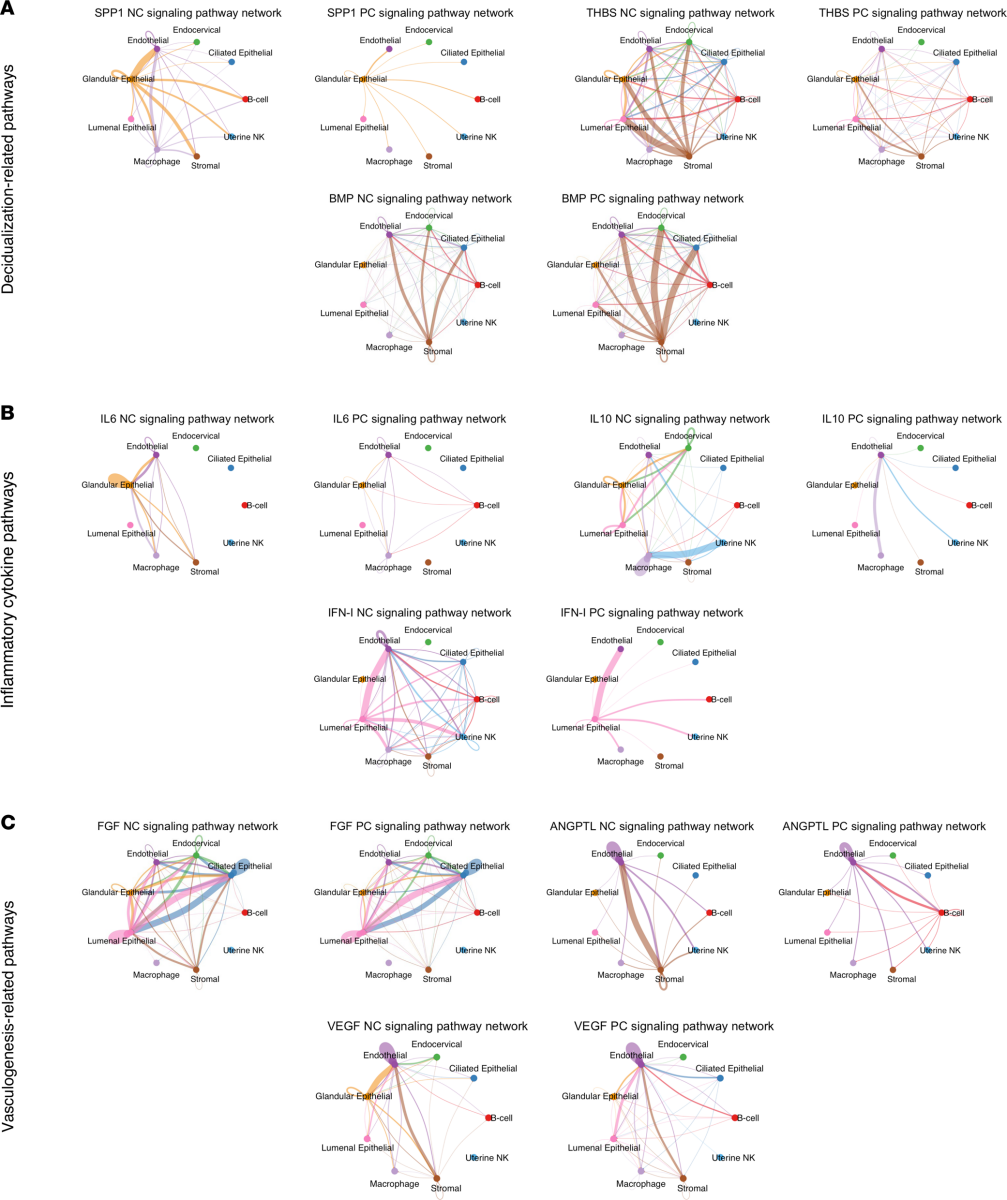
Cell-cell communication patterns of biological pathways relevant to reproduction by endometrial preparation method. (**A**) Circle plot visualization of inferred cell-cell communication network of pathways related to decidualization (SPP1, THBS, and BMP). (**B**) Circle plot visualization of inferred cell-cell communication network of pathways related to inflammatory cytokines (IL-10, IL-6, and INF-I). (**C**) Circle plot visualization of inferred cell-cell communication network of pathways related to angiogenesis and vascular remodeling (VEGF, FGF, and ANGPTL).

We further examined select pathways relevant to endometrial decidualization, inflammatory response, and angiogenesis between the 2 endometrial environments (Figure 4). The number of interactions in SPP1 and THBS pathways were decreased in PC compared with NC endometrium. On the other hand, BMP signaling was more prominent in PC endometrium. These observations highlight altered epithelial-stromal communication patterns that may affect decidualization and embryo implantation between endometrial preparation methods (Figure 4A). Signaling patterns were also altered in inflammatory pathways (IL-10, IL-6, and IFN-I), with less interaction numbers and strength in a PC microenvironment (Figure 4B). The complex interplay of these immunomodulators at the maternal-fetal interface may hold implications for maternal tolerance of the embryo as well as affecting trophoblastic invasion. Lastly, concordant with the functional enrichment analysis using stromal fibroblast DEGs, specific vasculogenesis/angiogenesis-related pathways were differentially signaled in NC versus PC (Figure 4C). Stromal fibroblast signaling in VEGF, ANGPTL, and FGF pathways appeared attenuated in PC endometrium. Together, these inferred cell-cell communication patterns suggest potentially perturbed signaling in key biological pathways relevant to reproduction and pregnancy success in a PC endometrial environment.

### Angiogenic balance in pregnancies from NC and PC endometrium.

We separately performed an independent, prospective clinical cohort study in 548 patients (Figure 1A; clinical metadata in Supplemental Table 3) to investigate our findings of potentially perturbed biological pathways related to decidualization and angiogenesis, as well as the postulated increased risk of HDP with PC endometrial preparation. Critically, we evaluated obstetric outcomes in pregnancies established in NC or PC endometrium while adjusting for ART use and health parameters related to underlying infertility — both of which could independently contribute to obstetric complications. This methodology helps pinpoint specific risk(s) attributed to endometrial preparation method.

**Figure 5:**
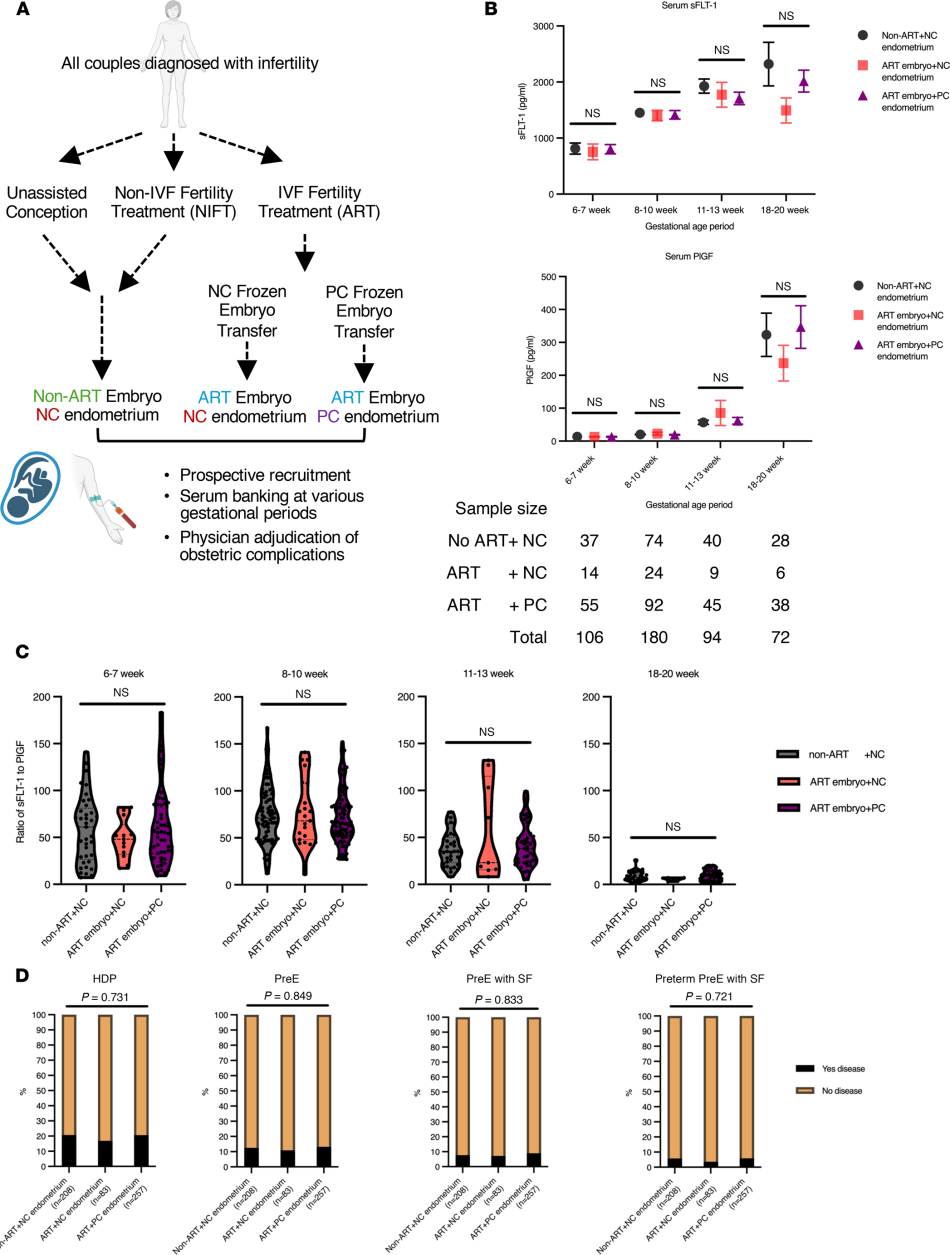
Angiogenic balance and incidences of hypertensive disorders of pregnancy by mode of conception. (**A**) Study design of a prospective clinical cohort to investigate the effect of PC endometrium on angiogenic balance and risk of HDP, adjusting for underlying infertility and use of ART. (**B**) Serum levels of angiogenic markers (sFlt1 and PlGF; mean ± SEM) by mode of conception across various gestational periods. Kruskal-Wallis test used. (**C**) Ratios of sFlt-1:PlGF by mode of conception and gestational period. Kruskal-Wallis test used. (**D**) Incidences of HDP (composite), preeclampsia (PreE), preeclampsia with severe features (PreE with SF), and preterm preeclampsia with severe features (Preterm PreE with SF; disease diagnosis < 37 weeks GA) by mode of conception (*P* value by χ^2^ test).

First, we examined serum levels of 2 maternal serum angiogenic markers, sFlt-1 and PlGF, in pregnancies conceived via 3 broad categories of mode of conception: (a) non-ART embryo implanted in a NC endometrium, (b) ART-derived embryo in a NC endometrium, and (c) ART-derived embryo in a PC endometrium (Figure 5A). We specifically measured early pregnancy levels of these 2 analytes to see if angiogenic imbalance is observed in the initial stages of establishing the maternal-fetal interface. As shown in Figure 5B., sFlt-1 concentrations did not significantly differ by mode of conception in early gestation, from 6–7 weeks through 18–20 weeks (all *P* > 0.05). Similarly, PlGF concentrations also did not differ significantly by mode of conception, regardless of ART use or endometrial preparation method. We then graphed serum sFlt-1/PlGF ratios to probe the state of angiogenic balance in these gestational periods (Figure 5C). Serum sFlt-1/PlGF ratios did not significantly differ by mode of conception at 6–7 weeks, 8–10 weeks, 11–13 weeks, or 18–20 weeks (all *P* > 0.05). sFlt-1/PlGF ratios were high in very early pregnancy but gradually and substantially shifted toward lower ratios by 18–20 weeks. This is likely secondary to a marked increase in PlGF at 18- to 20-week gestational age (GA) in all modes of conception (Figure 5B).

### Incidences of HDP by mode of conception.

Incidences of HDP diagnoses were subsequently analyzed in this prospective cohort of 548 singleton pregnancies: 208 conceived without ART in a NC endometrium (non-ART+NC), 83 conceived with an ART-derived embryo in a NC endometrium (ART+NC), and 257 conceived with an ART-embryo in a PC endometrium (ART+PC). We focused on specific diagnoses along the spectrum of this clinical syndrome: any HDP (which includes gestational hypertension), preeclampsia, preeclampsia with severe features, and preterm preeclampsia with severe features (defined as diagnosis at < 37 weeks GA). All diagnoses were adjudicated by OB/GYN-trained clinicians using the criteria set forth by the American College of Obstetricians and Gynecologists (47).

Baseline characteristics of this cohort are provided in Supplemental Table 3. Female patients who conceived with ART+NC or ART+PC were significantly older than those who conceived via non-ART+NC. The median (interquartile range [IQR]) age of female patients was 39 (range, 36–43) in ART+PC, 38 (range, 35–40) in ART+NC, and 36 (range, 34–38) in non-ART+NC (*P* < 0.01). Distribution of primarily infertility diagnoses was also significantly different among the 3 groups (*P* < 0.01). The proportion of nulliparity was significantly different, with the non-ART+NC group having the highest percentage of nulliparous patients (71.2%) (*P* < 0.01). The distribution of body mass index (BMI), history of prior miscarriage, and history of hypertension or diabetes diagnoses prior to pregnancy did not differ among the 3 groups (all *P* > 0.10).

We compared the incidences of various HDP diagnoses by mode of conception (Figure 5D). Composite HDP incidences did not differ significantly by mode of conception: 20.7% in non-ART +NC, 16.9% in ART+NC, and 20.6% in ART+PC (*P* = 0.73). We then analyzed if HDP diagnoses of greater severity, or earlier manifestation, would differ by mode of conception. The incidence of preeclampsia was 12.5% in non-ART+NC, 10.8% in ART+NC, and 13.2% in ART+PC (*P* = 0.85). Preeclampsia with severe features developed in 7.7% of patients in non-ART+NC, 7.2% in ART+NC, and 8.9% in ART+PC (*P* = 0.83). Lastly, the incidence of preterm preeclampsia with severe features were 5.8% in non-ART+NC, 3.6% in ART+NC, and 5.8% in ART+PC (*P* = 0.72). ART+PC also had similar rates of hypertensive disorders as patients in the non-ART+NC group. Finally, multivariable log-binomial regression was performed to estimate adjusted relative risk and 95% CI of HDP diagnoses by mode of conception, adjusting for age, BMI, nulliparity, and primary infertility diagnosis. Compared with non-ART+NC conceptions, there was no significantly increased RR of any of the HDP diagnoses in either ART+NC or ART+PC conceptions (Supplemental Table 4).

## Discussion

Our study compared the NC and PC WOI endometrium transcriptomic landscape at a single-nucleus resolution and contextualized the molecular differences using a prospective clinical study to investigate the associations between endometrial preparation method and pregnancy angiogenic balance as well as HDP risk. While endometrial preparation method in FET has been investigated in clinical studies, the debate remains on which preparation is superior (or if they are equivalent), and major knowledge gaps exist in how these endometrial preparation methods differ on a basic, cell type–specific level (4–6, 10, 11). Our findings demonstrate significant molecular differences in WOI endometrium by endometrial preparation method, with PC regimen associated with transcriptomic changes relevant to reproduction, including pathways involved in endometrial decidualization, angiogenesis, extracellular matrix remodeling, and the peri-implantation immune environment. Despite these transcriptomic differences, in an independent prospective clinical cohort adjusting for underlying infertility and relevant baseline risk factors, we found no significant associations between PC FET and angiogenic imbalance or HDP risk.

We first focused on key differences in transcriptomic landscape, cell-cell communication, and cellular topography between NC and PC endometrium. We showed that WOI transcriptomes, particularly in glandular epithelial and stromal fibroblast populations, are distinct between NC and PC endometrium. Our findings are concordant with a recent integrated single-cell endometrial atlas study by Marečková et al., which showed that exogenous hormones affect global gene expression in cells particularly from epithelial and mesenchymal lineages (26). However, in that study, the samples were largely exposed to contraceptive hormones and not those encountered in the context of normal ovulation or fertility treatment. Therefore, our study adds foundational data for a clinically relevant setting and time point that is critical for those undergoing IVF treatment as well as further understanding requirements for normal implantation. Functional enrichment and cell-cell interactome analyses showed possible perturbations on processes involving embryo implantation, angiogenesis, and extracellular matrix remodeling, primarily with downregulation of these pathways and/or decreased signaling in PC endometrium. PC endometrium also demonstrated higher epithelial/stromal ratio, which may have functional implications associated with architectural changes in key early tissue compartments for implantation. Since the inception of PC endometrial preparation, many have questioned how this protocol compares to NC in the clinical setting (4, 5). Previous randomized control trials suggested similar live birth rates between the 2 endometrial preparation methods (6, 48). However, the debate continues as the predetermined sample size was not achieved, overall live birth rates were lower than expected, and/or the ploidy status of the embryo transferred was not ascertained in these earlier clinical studies.

Based on our results, it is plausible to hypothesize that NC may provide a more optimal environment in the WOI during embryo transfer. Canonical epithelial and stromal receptivity markers appeared more highly expressed in a NC setting (Figure 2D and Supplemental Figure 3). Cell-cell communication patterns of different pathways involved in decidualization, tissue homeostasis, and inflammatory signaling were also attenuated in PC endometrium (Figure 4). Pathway analyses also suggest dysregulation of oxidative stress response in PC WOI endometrium, with significant downregulation of several key antioxidant enzymes (*GPX3*, log_2_FC –5.68; *GPX4*, log_2_FC 1.68; *SOD2*, log_2_FC –3.13; *SOD3*, log_2_FC 1.22; *SESN3*, log_2_FC –1.21; all *P*_adj_ < 0.0001). Alterations in these biological processes may predispose to an increased risk of HDP, as heightened oxidative stress and associated inflammatory response have been implicated in placental vascular dysfunction and HDP pathophysiology (49, 50). Uterine NK cells were also significantly less abundant in PC endometrium during the WOI, potentially due to decreased *CXCL14* expression and subsequently reduced chemokine signaling (Figure 2C and Figure 4B). The decrease in uterine NK cells in PC endometrium during the WOI may also favor NC endometrium clinically, as this specialized cell type has been proposed to optimize trophoblastic invasion, spiral artery remodeling, and placentation (51, 52). Of note, the most recent randomized control trial on this topic by Liu et al. compared outcomes in over 900 patients and demonstrated higher live birth rates, as well as lower miscarriage and antepartum hemorrhage rates in patients who underwent NC compared with PC FET (13). Our transcriptomic findings may, therefore, hold clinical relevance regarding endometrial optimization in IVF treatment and will inform future mechanistic studies. This dataset also provides insights that will complement future clinical studies to elucidate the relative importance of various endometrial markers and cell types in human implantation.

Given the clinical concern for increased HDP risk with PC FET (5, 19), we conducted a prospective clinical study to deeply investigate clinical angiogenic balance under different endometrial environments. Our cohort design addresses some critical limitations in previous studies by adjusting for baseline infertility status, preconception risk factors for HDP, and use of ART (10, 11). Despite the observed molecular differences in PC, sFlt-1 and PlGF levels were not significantly different by ART status or endometrial preparation method (Figure 5B). We also analyzed sFlt-1/PlGF ratios to probe early pregnancy angiogenic balance. We observed the angiogenic pendulum in human pregnancy to shift from a primarily antiangiogenic state in very early GAs to a proangiogenic state by the end of second trimester (Figure 5C). This represents one of the largest human datasets on angiogenic balance in early gestation, particularly in patients with infertility. Our findings are concordant with a foundational study by Levine et al. that profiled circulating angiogenic factors in fertile, healthy female patients (53). When analyzed by endometrial preparation method, sFLt-1/PlGF ratios were not significantly different by mode of conception in all GA periods through 20 weeks. These observations suggest that PC endometrial preparation alone do not result in clinical angiogenic imbalance at the maternal-fetal interface in early gestation. Combining our angiogenic marker findings with prior studies demonstrating very low or undetectable relaxin levels in PC-conceived pregnancies (15, 54), we postulate that very early redundant pathways at the endometrial level and/or embryo-derived factors independent of relaxin may compensate for differences observed between NC and PC WOI endometrium. Furthermore, we also did not observe significant differences in proportions of other placentation-related adverse outcomes (placental abruption, placenta previa, fetal growth restriction, and gestational diabetes) by mode of conception in our clinical cohort (Supplemental Table 5). Our observations support the notion that embryos are highly adaptable to varied endometrial environments, and/or that the endometrium has redundant mechanisms that buffer modest perturbations, thereby preserving the vital processes of early implantation and placentation in human reproduction.

Lastly, we assessed for HDP diagnoses under different modes of conception with rigorous physician adjudication. We found that the composite HDP incidence were not significantly different by endometrial preparation method (Figure 5D). We also evaluated specific diagnoses of varying levels of clinical severity (preeclampsia, preeclampsia with severe features, and preterm preeclampsia with severe features) and found no significant difference. A post hoc power calculation, using the same baseline assumptions as an ongoing trial (55) designed to demonstrate differences in preeclampsia risk, showed that our live birth sample size provides > 80% power to detect an 8.3% difference in preeclampsia risk between NC and PC pregnancies. The recent randomized control trial by Liu et al. also did not demonstrate significant differences in HDP risk between NC FET (*n* = 248) and PC FET (*n* = 201) live births (13). Lastly, another retrospective study also showed no increased risk of ischemic placental disease (including preeclampsia) comparing PC with NC FET pregnancies in over 800 females (12). It is worth noting that patients with infertility who conceived without ART also had higher HDP risk compared with the general population based on historical data (56), echoing that patients with infertility are at a higher baseline risk regardless of treatment modality. One possible explanation for the discordant findings between our study and others is that the effects of an absent corpus luteum are minor relative to the underlying infertility and become unobservable after adjusting for baseline risk factors such as older age and nulliparous status.

Our findings question the notion that the corpus luteum and its associated products have a substantial protective effect against HDP in frozen embryo transfer pregnancies and suggest that HDP risk is largely attributed to an individual’s preexisting health status and obstetric history. Until proven by high-quality clinical evidence (55), our study suggests that clinicians should not recommend NC endometrial preparation for the sole purpose of reducing HDP risk. Patients who require PC endometrial preparation to optimize embryo transfer should not be subjected to unnecessary stress based on the assumption that they are at increased risk for adverse obstetric outcomes.

This study has several key strengths. First, we rigorously selected and sampled patients at matched WOI time points following detailed clinical evaluation, excluding individuals with concomitant uterine/endometrial pathology that could confound gene expression. All samples were obtained using an identical technique by the same proceduralist. These measures allow us to more precisely isolate the effects of hormonal modulation, reducing the likelihood of detecting differences related to procedural technique or infertility-associated conditions. Single-nucleus sequencing studies are also susceptible to batch effects. Therefore, we employed single-batch processing under uniform experimental conditions and sequencing parameters. We also correlated our transcriptomic findings with an independent clinical cohort representative of the patient population treated within the same healthcare system. Lastly, HDP outcomes were adjudicated by OB/GYN-trained clinicians with expertise in diagnosing and identifying obstetric complications, increasing confidence in outcome reporting and overall study validity.

We also acknowledge limitations of this study. First, our samples and analyses focused on WOI endometrium at a single, defined time point to better understand what the embryo “sees” when placed in the uterine cavity during IVF. Although the number of processed samples is smaller than some prior studies (26, 42), it represents the maximum feasible per batch with our experimental methodology, which was designed to satisfy general requirements for RNA-seq experiments and minimize batch effects. Differences in transcriptomic profiles may also not correlate fully with proteomic changes or the functional state of the cells. Moreover, the sample size of the clinical cohort is also smaller than those of registry- or population-based studies, which may limit the power to detect subtle differences in HDP risk. Our study population was also of relatively lean BMI with limited diversity, potentially limiting our ability to identify increased HDP risk associated with PC endometrial preparation. Nonetheless, this remains the largest prospective clinical cohort to date examining the isolated effects of ART on obstetric complications. This study design reduces heterogeneity, enhances the granularity and accuracy of both exposure and outcome data, and retains sufficient power to detect clinically meaningful increases in adverse outcomes.

Future studies that assess all major time points of the menstrual cycle, under different endometrial preparation methods with extensive clinical metadata and analysis of confounders, will further delineate how these endometrial environments differ. Conclusions about implantation, decidualization, and placentation processes cannot be definitively drawn from the perspective of the endometrium alone without also considering the embryo — and vice versa. This is particularly challenging to study in humans, especially those experiencing infertility. Embryo-endometrium crosstalk also likely plays a critical role and may enhance our understanding of both normal and pathological conditions in reproductive medicine (57). Future in vitro studies combining human endometrial assembloids and blastoids offer promising potential to improve our understanding and modeling of the peri-implantation “black box” period of human reproduction (58, 59).

In summary, we generated a single-cell atlas of PC endometrium to define cell type–specific differences relative to NC endometrium. We found that PC endometrial preparation markedly alters molecular signatures, particularly in glandular epithelial and stromal fibroblasts, potentially resulting in perturbations in implantation, decidualization, and placentation pathways. Cellular composition of PC endometrium also differs markedly from NC, with a striking reduction in uterine NK cells. Despite these changes, our findings did not translate to subsequent angiogenic imbalance or increased HDP risk in a rigorously examined prospective infertile patient cohort. These results suggest that the maternal-fetal interface in early gestation is highly adaptable and that potential deficiencies associated with PC endometrium alone are unlikely to confer significant clinical risk for HDP.

## Methods

### Sex as a biological variable

We only examined tissue and outcomes from female patients as the disease modeled (pregnancy) is only relevant for females.

### WOI endometrium samples

We prospectively sampled patients who are facing infertility and was ruled out for uterine/endometrial abnormalities in NC and PC conditions. All samples (*n* = 16) were obtained using an endometrial Pipelle catheter (CooperSurgical). NC WOI biopsies were obtained on the seventh day following endogenous luteinizing hormone surge with no exogenous medications. PC WOI biopsies were obtained on the sixth day of progesterone-in-oil administration. The medication regimens for PC patients are summarized in Supplemental Table 2; there was no pharmacologic pituitary downregulation prior to exogenous hormone administration. All biopsies were performed by the same physician to limit inter-proceduralist variability. Tissue samples were immediately frozen in liquid nitrogen and stored at –80°C.

### Nuclei isolation and snRNA-seq

#### Nuclei suspension.

Single nuclei suspensions were obtained using the Singulator 100 system (S2 Genomics). Tissues were slightly thawed and transferred to nuclei isolation cartridges with 30uL of RNase inhibitor. Nuclei isolation followed the Singulator Low Volume Nuclei Isolation v2 preprogrammed protocol. Suspensions were centrifuged at 500g x5minutes at 4C. Resultant pellets were washed 1x with ice-cold wash buffer (0.4% BSA in PBS). Washed pellets were resuspended in ice-cold wash buffer and filtered through a 40uM FlowMi cell strainer (#136800040,Sigma-Aldrich). Nuclei count of each suspension was obtained using CellacaMX.

#### Nuclei fixation.

The 10x Genomics protocol for nuclei fixation was performed (CG000478). Nuclei suspensions were centrifuged at 500g x5minutes at 4C and resuspended in freshly prepared Fixation Buffer, which contains 4% formaldehyde (252549-500ML, Sigma-Aldrich), nuclease free water (BP2819-1,Fisher Bioreagents), and 1x Concentrated Fix and Perm Buffer (PN-2000517,10x Genomics). Samples were fixed at 4°C for 23 hours.

#### Postfixation quenching and probe hybridization.

After 23-hour fixation, nuclei were pelleted by centrifuging the suspensions at 850*g* for 5 minutes. Pellets were resuspended in 1 mL of Quench buffer (nuclease free water and 1x Concentrated Quench Buffer; PN-2000516,10x Genomics). Samples were counted again post-fixation. Nuclei were hybridized at 42°C for 20 hours with whole transcriptome probe pairs designed for mRNA targets (Human Transcriptome Probe Kit,PN-1000475).

#### Single-nucleus encapsulation, library construction, and sequencing.

Probe hybridization reaction was quenched, and an equal number of nuclei were pooled from each sample. Each sample was labeled with unique probe barcodes to allow multiplexing of all samples into a single batch using the 10x Chromium Fixed RNA kit. The pooled nuclei suspension was washed to remove unbound probes, and 250,000 nuclei were loaded onto the Chromium Next GEM Chip Q. The Chromium X instrument was used to generate Gel Beads-in-Emulsion (GEMs). Within each GEM, a copartitioned nucleus was lysed to allow Gel Bead primer hybridization with the sequence on ligated probes. This hybridization incorporated a 16-nucleotide GEM barcode and a 12-nucleotide unique molecular identified (UMI). The Recovery Agent was used to break the GEMs, and resultant barcoded products were amplified by PCR and purified with SPRI-select beads. A final PCR step was performed to add Illumina TruSeq adapters (P5 and P7) and i5/i7 indices for multiplexing (Dual Index Kit TS Set A, PN-1000251). Sequencing was conducted on the Illumina NovaSeqX-25B at a target depth of 20,000 reads per cell with 5% PhiX spike-in for diversity control.

### SnRNA-seq data analysis

#### Data processing.

Raw reads from FASTQ files were demultiplexed using unique probe barcodes, aligned to the human reference transcriptome (GRCh38-2020-A), and counted using Cell Ranger multi Software (60). Filtered reads were individually loaded into Seurat (61). Ambient RNA correction was performed with CellBender using default parameters (62). Initial quality control of Seurat objects was performed by removing nuclei with < 200 genes and genes expressed in < 3 cells. The filtered Seurat objects were merged, and the combined dataset was further filtered to remove low-quality nuclei, empty droplets, and doublets. Nuclei with > 3% mitochondrial reads, < 350 or > 7,500 genes, or UMI count > 30,000 were removed. The dataset was processed using a standard single cell workflow, with log-normalization and scaling using the *NormalizeData* and *ScaleData* Seurat functions (63). Effects related to cell cycle phase and percentage of mitochondrial reads were regressed out. Principal component analysis (PCA) was performed using input from the *FindVariableFeatures* function (2,000 variable features). The top 30 PCs were used as input to construct a nearest neighbor graph using *FindNeighbors*, and Louvain clustering with *FindCluster* at resolution 0.5. UMAP nonlinear dimensional reduction was used to visualize clusters. Marker genes for the clusters were identified using *FindAllMarkers* with default parameters; clusters with high expression of mitochondrial genes or nonspecific expression were removed. After removal of nonspecific and high mitochondrial content clusters, the process of variable gene selection, data scaling, PCA, and clustering/UMAP was repeated to obtain the final dataset.

#### Annotation and subclustering.

Cell type markers proposed by existing literature (9, 26, 64, 65) were used to perform annotation based on most highly expressed genes and relative expression of canonical cell type markers in each cluster. Clusters from major cell types were further individually subclustered, reintegrated, and filtered with the process described above to remove low-quality nuclei. Canonical cell type markers by annotated clusters are shown in Supplemental Figure 1.

#### Differential abundance analysis.

Crude percentages of cell types by endometrial preparation condition were presented in a bar graph. We used MiloR (29), which performs differential abundance testing by grouping cells into neighborhoods with *k-*nearest neighbor (KNN) graph (*k* = 20, *d* = 30), to analyze differential abundance of cell populations. A beeswarm plot was used to depict the distribution of log_10_FC of neighborhoods containing each individual cell type in NC and PC endometrium.

#### Differential gene expression and gene ontology enrichment analysis.

Data were pseudobulked using *AggregateExpression*. DEG analysis was conducted using the *FindMarkers* function with DESeq2 (66). The number of significant up/downregulated DEGs (log_2_FC > 0.1 and *P*_adj_ < 0.05) by cell type is shown in a bar graph. Volcano plots were generated using the EnhancedVolcano package (67). DEGs were used as input for GSEA of Gene Ontology Biological Process gene sets using the clusterProfiler package (68). Ridgeline plots were generated to visualize distributions of gene expression for GSEA-enriched categories.

#### Cell-cell interactome.

Cell-cell communication was inferred using CellChat and the CellChatDB.human database (38). Overexpressed genes/interactions were identified using *identifyOverExpressedGenes* and *identifyOverExpressedInteractions*, and communication probabilities were calculated within each condition (NC, PC) using *computeCommunProb*. This was followed by *computeCommunProbPathway* to predict signaling pathways and their relative strengths. CellChat objects were merged to facilitate signaling comparisons of different cell groups and pathways between conditions. Number of interactions and interaction strengths of aggregated communication networks were visualized using circle plots. The information flow of a pathway, reflecting both the extent and strength of communication, was calculated using the sum of communication probabilities across all ligand-receptor pairs of interacting cell types. Relative information flow in NC and PC was computed using *rankNet* to inform global differences in cell-cell communication pathways between the 2 conditions, which facilitated specific pathways for subsequent circle plot visualization.

### Prospective clinical cohort for obstetric complication assessment

An independent, prospectively recruited cohort was drawn from the Developmental Epidemiological Study of Children born through Reproductive Technologies (DESCRT) study at UCSF to correlate clinical outcomes in patients who conceived under NC or PC endometrium based on our transcriptomic findings from the snRNA-seq cohort (69). One of its main aims is to examine the effects of ART on the intrauterine environment, focusing on obstetric/perinatal outcomes. Details of enrollment and study protocol were previously published (69). In this study, a total of 548 singleton pregnancies from patients diagnosed with infertility were reviewed and analyzed: 208 conceived without ART in a NC endometrium, 83 with an ART-embryo in a NC endometrium, and 257 with an ART-embryo in a PC endometrium. ART conceptions are those that used oocytes and/or embryos that derived from oocyte retrieval and in vitro culture/manipulation. This unique cohort and methodology help account for effects from (a) underlying infertility, (b) ART, and (c) baseline health-related risk factors, thereby isolating the putative effects of endometrial preparation on HDP risk. Multiple gestations were excluded due to their known higher risk of HDP. Baseline parental characteristics and covariates were systematically ascertained (Supplemental Table 3). Mode of conception and ART parameters are rigorously tracked as part of routine clinical care and laboratory quality control.

### Maternal serum angiogenic markers

Serum was obtained at specific GA periods when available (6–7, 8–10, 11–13, and 18–20 weeks GA) and stored at –80°C. Not all participants could contribute to the serum bank due to COVID-19 restrictions during the study period. Serum sFlt-1 and PlGF were measured. A validated, FDA-approved electrochemiluminescence immunoassay was used to measure both analytes (Roche Diagnostics, Switzerland). These immunoassays were compatible with and performed on the Roche Diagnostics Elecsys cobasâ analyzer, a fully automated system used for clinical diagnostics at UCSF. The detection range was 10–85,000 pg/mL for sFlt-1 and 3–10,000 pg/mL for PlGF. Duplicate measurements were performed on 20% of the dataset to ensure inter- and intra-assay coefficient of variation of less than 8%.

### HDP

The primary outcome for the clinical cohort study was the composite incidence of HDP, which encompasses gestational hypertension, preeclampsia without severe features (preE), preeclampsia with severe features (preE with SF), and/or eclampsia. Pregnancy trajectories, obstetric records, and postpartum encounters were systematically reviewed, and HDP diagnoses were adjudicated by OB/GYN-trained clinicians using criteria set forth by the American College of Obstetricians and Gynecologists guidelines (47). GA at which the HDP diagnosis was made was also ascertained. Preterm preeclampsia was defined as disease diagnosis prior to 37 weeks GA.

### Statistics

For DEG, GSEA, and cell-cell communication analyses, *P* values were adjusted using the FDR with Benjamini-Hochberg correction for multiple testing. *P*_adj_ < 0.05 was considered statistically significant. For miloR differential abundance analysis, a spatial FDR threshold of < 0.1 was used, followed by a binomial test to examine differences in number of enriched neighborhoods across conditions (threshold *P* < 0.05). Statistical analyses for snRNA-seq data were conducted using R. For comparisons of clinical variables among mode of conception groups, the Kruskal-Wallis test, χ^2^ test, or Fisher’s exact test was used as appropriate. Mean ± SEM values were graphed for sFlt-1 and PlGF concentrations. Kruskal-Wallis test was used to compare the distributions of sFlt-1/PlGF concentrations and sFlt-1/PlGF ratios. HDP incidences by mode of conception were compared using the χ^2^ test. Univariable and multivariable log-binomial regression were used to generate crude and adjusted risk ratios and 95% CI to assess for associations between mode of conception and HDP diagnoses. For clinical cohort analyses, all tests were 2-sided and conducted at the 0.05 level of significance using Stata v17.

### Study approval

Endometrial tissue banking was approved by UCSF IRB 10-02786. The UCSF DESCRT study was approved by UCSF IRB 16-20474 and registered on ClinicalTrials.gov (NCT03799107).

### Data availability

Processed snRNA-seq data are deposited in the CellxGene Discover (https://cellxgene.cziscience.com/collections/e80f5450-0a0f-4312-a728-431d50aeb8fe). R codes are publicly accessible on GitHub (https://github.com/huangdav/natproendometrium; commit ID 74fc2c3). Values for data points associated with applicable graphs and reported means are included in the Supporting Data Values file. The clinical cohort dataset contains potentially identifiable information and will be made available from the DESCRT study team investigators (DH, MIC) upon request and execution of a data transfer agreement.

## Author contributions

Study design was contributed by DH, BD, JCI, MN, MPR, LBZ, GKF, AJC, MS, MIC, and LCG. Experiments were conducted by DH, BD, JCI, MN, YS, FBR, and RW. Data were acquired by DH, EF, BD, MN, ALA, JQ, YS, RW, and MS. Analyzing data was contributed by DH, EF, BD, LBZ, GKF, AJC, and MIC. Reagents were provided by BD, JCI, FBR, MPR, TW, AJC, MIC, and LCG. DH, EF, and BD wrote the original draft. DH, EF, BD, JCI, ALA, JQ, YS, FBR, RW, LBZ, MPR, GKF, AJC, MS, MIC, and LCG reviewed and edited the manuscript.

## Conflict of interest

The authors have declared that no conflict of interest exists.

## Funding support

This work is in part the result of NIH funding, and is subject to the NIH Public Access Policy. Through acceptance of this federal funding, the NIH has been given a right to make the work publicly available in PubMed Central.

- ABOG/AAOGF Award to DH
- NICHD R01 HD084380 to MIC and LBZ
- NCTRI P50 HD055764 to LCG and MS
- PREMIER (NIAMS P30AR070155-05) and the Bakar ImmunoX Initiative to EF and GKF

## Supplementary Material

Supplemental data

ICMJE disclosure forms

Supporting data values

## Version 1 06/23/2026

In-Press Preview

## Version 2 08/10/2026

Electronic publication

## References

[1] European IVF Monitoring Consortium (EIM) for the European Society of Human ReproductionEmbryology (ESHRE) (2023). ART in Europe, 2019: results generated from European registries by ESHRE†. Hum Reprod.

[2] https://stacks.cdc.gov/view/cdc/158671.

[3] Hipp HS (2022). Trends and outcomes for preimplantation genetic testing in the United States, 2014-2018. JAMA.

[4] Groenewoud ER (2018). Programming the endometrium for deferred transfer of cryopreserved embryos: hormone replacement versus modified natural cycles. Fertil Steril.

[5] Wu H (2021). Endometrial preparation for frozen-thawed embryo transfer cycles: a systematic review and network meta-analysis. J Assist Reprod Genet.

[6] Ho VNA (2024). Live birth rate after one frozen embryo transfer in ovulatory women starting with natural, modified natural, or artificial endometrial preparation in Viet Nam: an open-label randomised controlled trial. Lancet.

[7] Pirtea P (2023). Recurrent implantation failure: reality or a statistical mirage?: Consensus statement from the July 1, 2022 Lugano Workshop on recurrent implantation failure. Fertil Steril.

[8] Cao D (2025). Time-series single-cell transcriptomic profiling of luteal-phase endometrium uncovers dynamic characteristics and its dysregulation in recurrent implantation failures. Nat Commun.

[9] Wang W (2020). Single-cell transcriptomic atlas of the human endometrium during the menstrual cycle. Nat Med.

[10] Busnelli A (2022). Obstetric and perinatal outcomes following programmed compared to natural frozen-thawed embryo transfer cycles: a systematic review and meta-analysis. Hum Reprod.

[11] Zaat TR (2023). Obstetric and neonatal outcomes after natural versus artificial cycle frozen embryo transfer and the role of luteal phase support: a systematic review and meta-analysis. Hum Reprod Update.

[12] Farrell AS (2024). Perinatal outcomes are similar in programmed and modified natural frozen embryo transfer cycles. Reprod Biomed Online.

[13] Liu X (2025). Natural cycle versus hormone replacement therapy as endometrial preparation in ovulatory women undergoing frozen-thawed embryo transfer: the compete open-label randomized controlled trial. PLoS Med.

[14] Conrad KP (2022). Potential role of the corpus luteum in maternal cardiovascular adaptation to pregnancy and preeclampsia risk. Am J Obstet Gynecol.

[15] Conrad KP (2019). Potential influence of the corpus luteum on circulating reproductive and volume regulatory hormones, angiogenic and immunoregulatory factors in pregnant women. Am J Physiol Endocrinol Metab.

[16] von Versen-Höynck F (2019). Increased preeclampsia risk and reduced aortic compliance with in vitro fertilization cycles in the absence of a corpus luteum. Hypertension.

[17] Bryant-Greenwood GD (1993). Sequential appearance of relaxin, prolactin and IGFBP-1 during growth and differentiation of the human endometrium. Mol Cell Endocrinol.

[18] Goldsmith LT (2004). Relaxin regulation of endometrial structure and function in the rhesus monkey. Proc Natl Acad Sci U S A.

[19] Zaat TR (2021). Increased obstetric and neonatal risks in artificial cycles for frozen embryo transfers?. Reprod Biomed Online.

[20] Luke B (2017). Pregnancy and birth outcomes in couples with infertility with and without assisted reproductive technology: with an emphasis on US population-based studies. Am J Obstet Gynecol.

[21] Stern JE (2022). Assisted reproductive technology treatment increases obstetric and neonatal risks over that of the underlying infertility diagnosis. Fertil Steril.

[22] Stern JE (2022). Assisted reproductive technology or infertility: what underlies adverse outcomes? Lessons from the massachusetts outcome study of assisted reproductive technology. F S Rev.

[23] Maynard SE (2003). Excess placental soluble fms-like tyrosine kinase 1 (sFlt1) may contribute to endothelial dysfunction, hypertension, and proteinuria in preeclampsia. J Clin Invest.

[24] Berg AH (2026). Serum soluble fms-like tyrosine kinase and placental growth factor ratio on Elecsys immunoassay platform predicts preeclampsia with severe features in hospitalized women with hypertensive disorders of pregnancy. Am J Obstet Gynecol.

[25] Zeisler H (2016). Predictive value of the sFlt-1/PlGF ratio in women with suspected preeclampsia. N Engl J Med.

[26] Marečková M (2024). An integrated single-cell reference atlas of the human endometrium. Nat Genet.

[27] Admati I (2025). Single-nuclei RNA-sequencing fails to detect molecular dysregulation in the preeclamptic placenta. Placenta.

[28] Chemerinski A (2024). The impact of ovarian stimulation on the human endometrial microenvironment. Hum Reprod.

[29] Dann E (2022). Differential abundance testing on single-cell data using k-nearest neighbor graphs. Nat Biotechnol.

[30] Altmäe S (2017). Meta-signature of human endometrial receptivity: a meta-analysis and validation study of transcriptomic biomarkers. Sci Rep.

[31] Song H, Lim H (2006). Evidence for heterodimeric association of leukemia inhibitory factor (LIF) receptor and gp130 in the mouse uterus for LIF signaling during blastocyst implantation. Reproduction.

[32] Díaz-Gimeno P (2011). A genomic diagnostic tool for human endometrial receptivity based on the transcriptomic signature. Fertil Steril.

[33] Cheng JG (2001). Dual control of LIF expression and LIF receptor function regulate Stat3 activation at the onset of uterine receptivity and embryo implantation. Proc Natl Acad Sci U S A.

[34] Hu J (2024). Decreased thrombospondin-1 impairs endometrial stromal decidualization in unexplained recurrent spontaneous abortion†. Biol Reprod.

[35] Watanabe H (2005). A novel protein Depp, which is induced by progesterone in human endometrial stromal cells activates Elk-1 transcription factor. Mol Hum Reprod.

[36] Lu J (2016). CXCL14 as an emerging immune and inflammatory modulator. J Inflamm (lond).

[37] Mokhtar NM (2010). Progestin regulates chemokine (C-X-C motif) ligand 14 transcript level in human endometrium. Mol Hum Reprod.

[38] Jin S (2021). Inference and analysis of cell-cell communication using CellChat. Nat Commun.

[39] Tremaine TD, Fouladi-Nashta AA (2021). Steroid regulation of secreted phosphoprotein 1 (SPP1) expression in ovine endometrium. Reprod Fertil Dev.

[40] Guzeloglu-Kayisli O (2009). The role of growth factors and cytokines during implantation: endocrine and paracrine interactions. Semin Reprod Med.

[41] Viganò P (2002). Expression of interleukin-10 and its receptor is up-regulated in early pregnant versus cycling human endometrium. J Clin Endocrinol Metab.

[42] Yang N (2024). Trophoblastic signals facilitate endometrial interferon response and lipid metabolism, ensuring normal decidualization. Cell Rep.

[43] Brown N (2004). Embryo-uterine interactions via the neuregulin family of growth factors during implantation in the mouse. Biol Reprod.

[44] Monsivais D (2021). Endometrial receptivity and implantation require uterine BMP signaling through an ACVR2A-SMAD1/SMAD5 axis. Nat Commun.

[45] King AE (2001). Cd40 expression in uterine tissues: a key regulator of cytokine expression by fibroblasts. J Clin Endocrinol Metab.

[46] Hou T (2024). Semaphorin 4A maintains trophoblastic function via activating the STAT3 pathway. Biomolecules.

[47] [no authors listed] (2020). Gestational hypertension and preeclampsia: ACOG practice bulletin, number 222. Obstet Gynecol.

[48] Groenewoud ER (2016). A randomized controlled, non-inferiority trial of modified natural versus artificial cycle for cryo-thawed embryo transfer. Hum Reprod.

[49] Schoots MH (2018). Oxidative stress in placental pathology. Placenta.

[50] Redman CWG, Sargent IL (2003). Pre-eclampsia, the placenta and the maternal systemic inflammatory response--a review. Placenta.

[51] Huhn O (2021). How do uterine natural killer and innate lymphoid cells contribute to successful pregnancy?. Front Immunol.

[52] Moffett-King A (2002). Natural killer cells and pregnancy. Nat Rev Immunol.

[53] Levine RJ (2004). Circulating angiogenic factors and the risk of preeclampsia. N Engl J Med.

[54] Von Versen-Höynck F (2019). Effect of mode of conception on maternal serum relaxin, creatinine, and sodium concentrations in an infertile population. Reprod Sci.

[55] Baksh S (2021). Natural vs. programmed cycles for frozen embryo transfer: study protocol for an investigator-initiated, randomized, controlled, multicenter clinical trial. Trials.

[56] Ford ND (2022). Hypertensive disorders in pregnancy and mortality at delivery hospitalization - United States, 2017-2019. MMWR Morb Mortal Wkly Rep.

[57] Macklon NS, Brosens JJ (2014). The human endometrium as a sensor of embryo quality. Biol Reprod.

[58] Gantner CW (2025). Assembly of a stem cell-derived human postimplantation embryo model. Nat Protoc.

[59] Heidari Khoei H (2023). Generating human blastoids modeling blastocyst-stage embryos and implantation. Nat Protoc.

[60] Zheng GXY (2017). Massively parallel digital transcriptional profiling of single cells. Nat Commun.

[61] Stuart T (2019). Comprehensive integration of single-cell data. Cell.

[62] Fleming SJ (2023). Unsupervised removal of systematic background noise from droplet-based single-cell experiments using CellBender. Nat Methods.

[63] Butler A (2018). Integrating single-cell transcriptomic data across different conditions, technologies, and species. Nat Biotechnol.

[64] Garcia-Alonso L (2021). Mapping the temporal and spatial dynamics of the human endometrium in vivo and in vitro. Nat Genet.

[65] Fonseca MAS (2023). Single-cell transcriptomic analysis of endometriosis. Nat Genet.

[66] Love MI (2014). Moderated estimation of fold change and dispersion for RNA-seq data with DESeq2. Genome Biol.

[67] https://github.com/kevinblighe/EnhancedVolcano.

[68] Xu S (2024). Using clusterProfiler to characterize multiomics data. Nat Protoc.

[69] Adeleye AJ (2023). Study protocol for a developmental epidemiological study of children born through reproductive technologies (DESCRT). Hum Reprod Open.

