# Supplementary material for: Programmed cycle-induced endometrial perturbations do not independently influence angiogenic imbalance or hypertensive disorders in pregnancy
Source: JCI Insight. 2026 Jun 23;11(15):e202443. doi: 10.1172/jci.insight.202443 (PMC13463606; doi:10.1172/jci.insight.202443)
Supplement: Supplementary file 1 [file jciinsight-11-202443-s126.pdf]

SUPPLEMENTAL MATERIALS

Supplemental Figure S1.

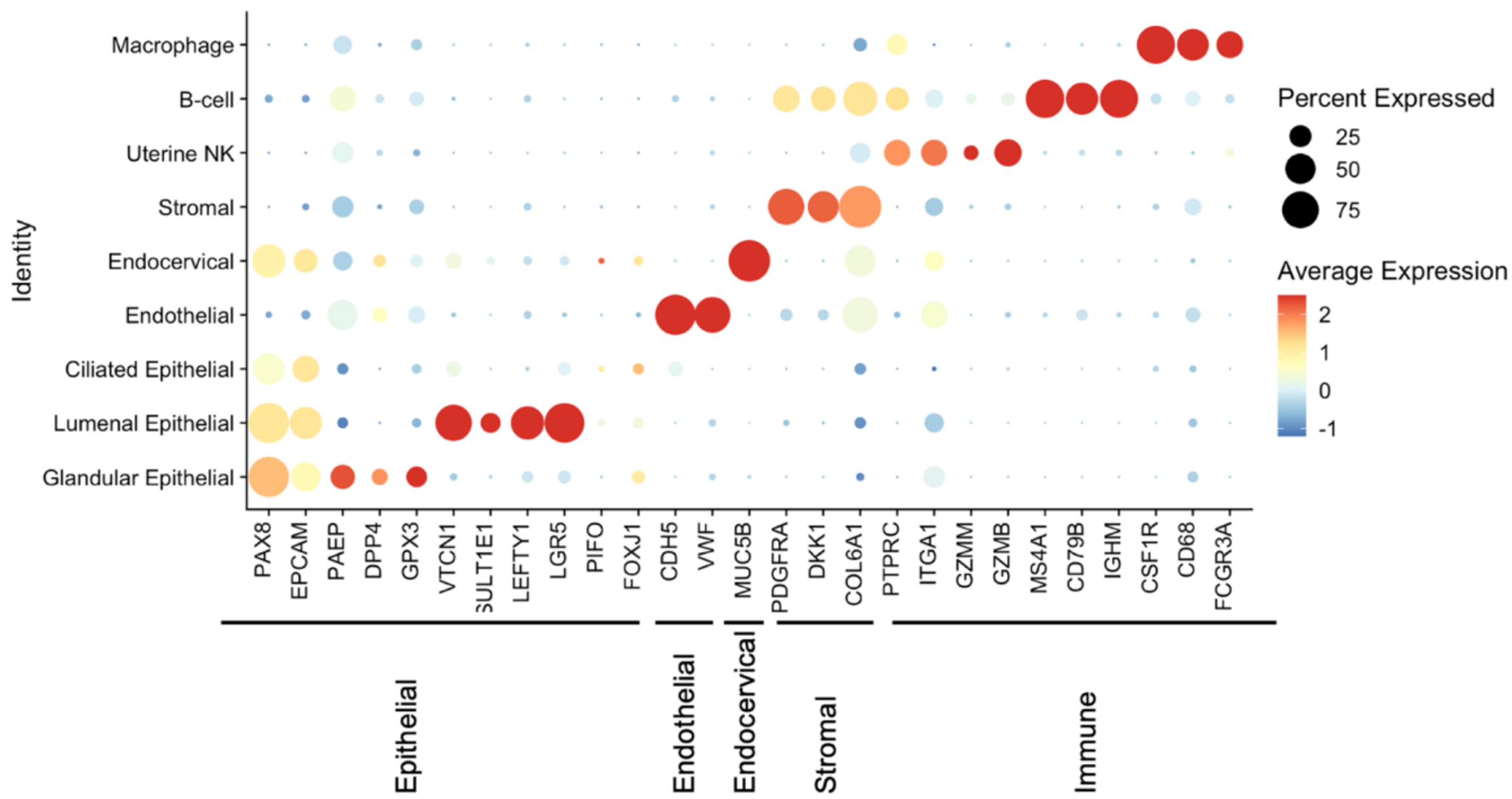

Supplemental Figure S1. Canonical cell type markers by annotated cell type clusters

Supplemental Figure S2.

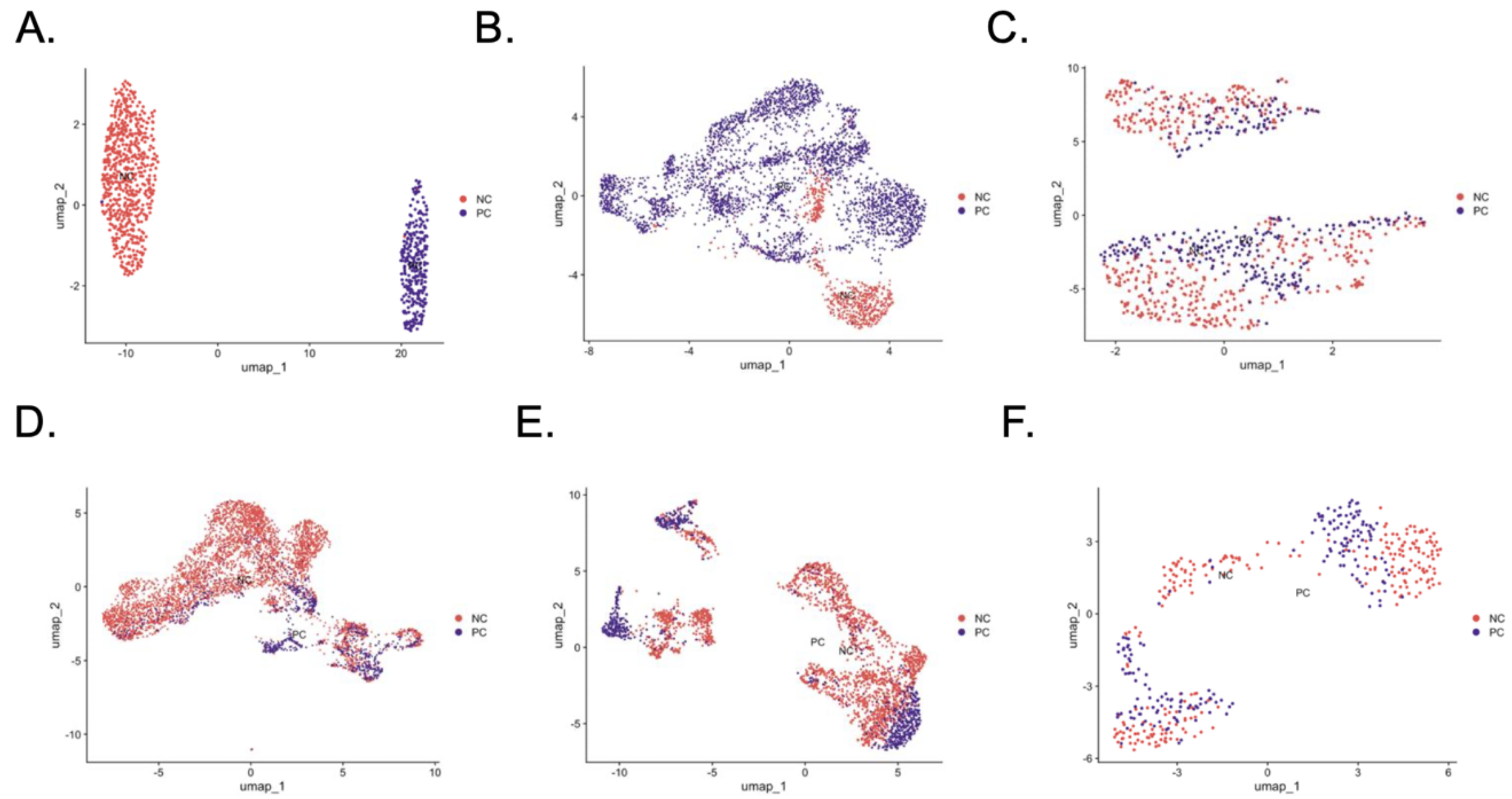

Supplemental Figure S2. UMAP of additional cell types by NC versus PC WOI endometrium. A) Ciliated Epithelial B) Luminal Epithelial C) Endothelial D) Uterine NK E) Macrophage F) B cell

Supplemental Figure S3.

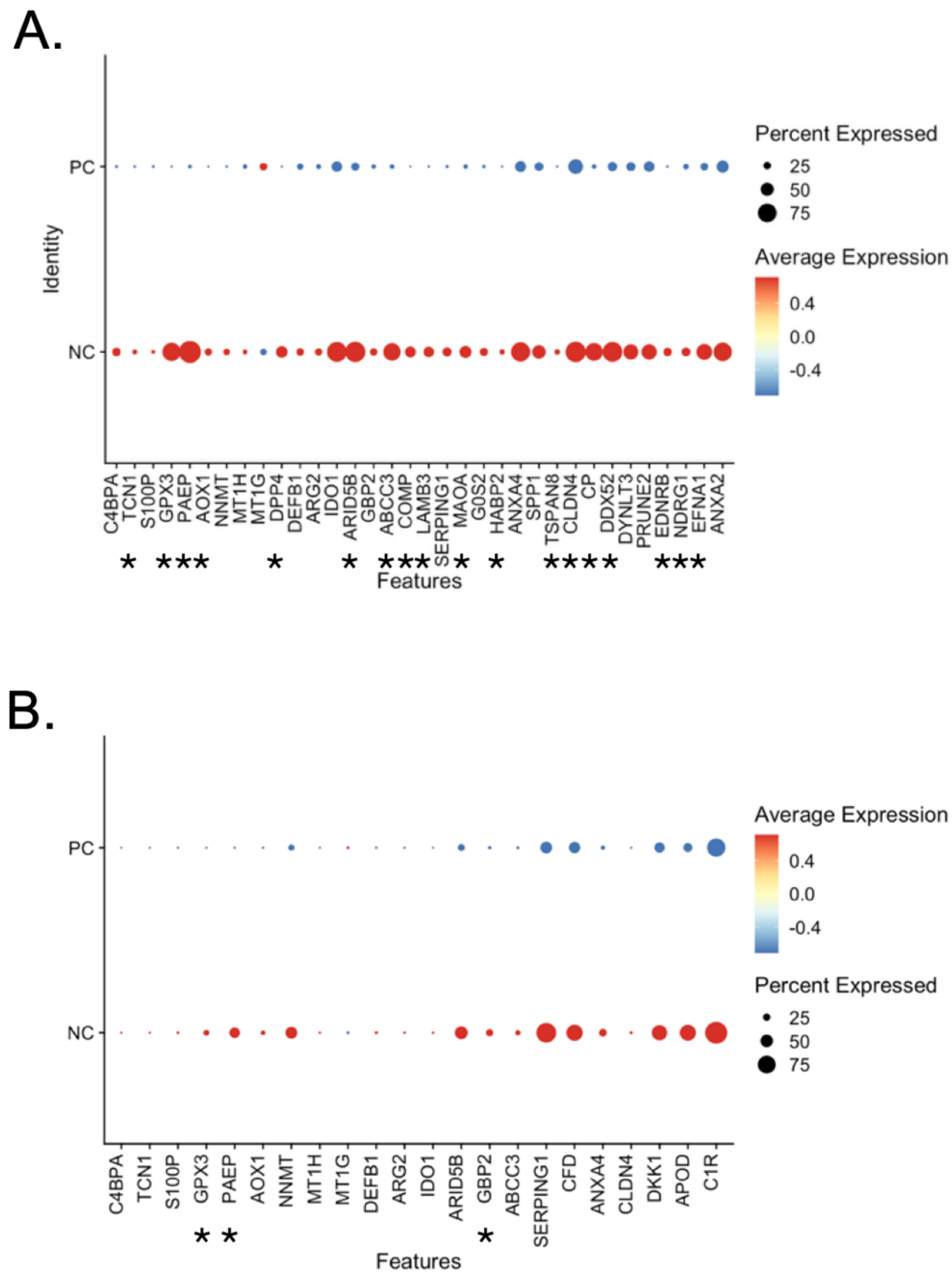

Supplemental Figure S3. Dot Plot of relative expression of canonical endometrial receptivity markers in NC versus PC endometrium. A) Epithelial-specific receptivity markers **B)** Stromal fibroblast-specific receptivity markers. Statistically significance (adjusted p-value <0.05; DESeq2) is indicated by (*).

Supplemental Figure S4.

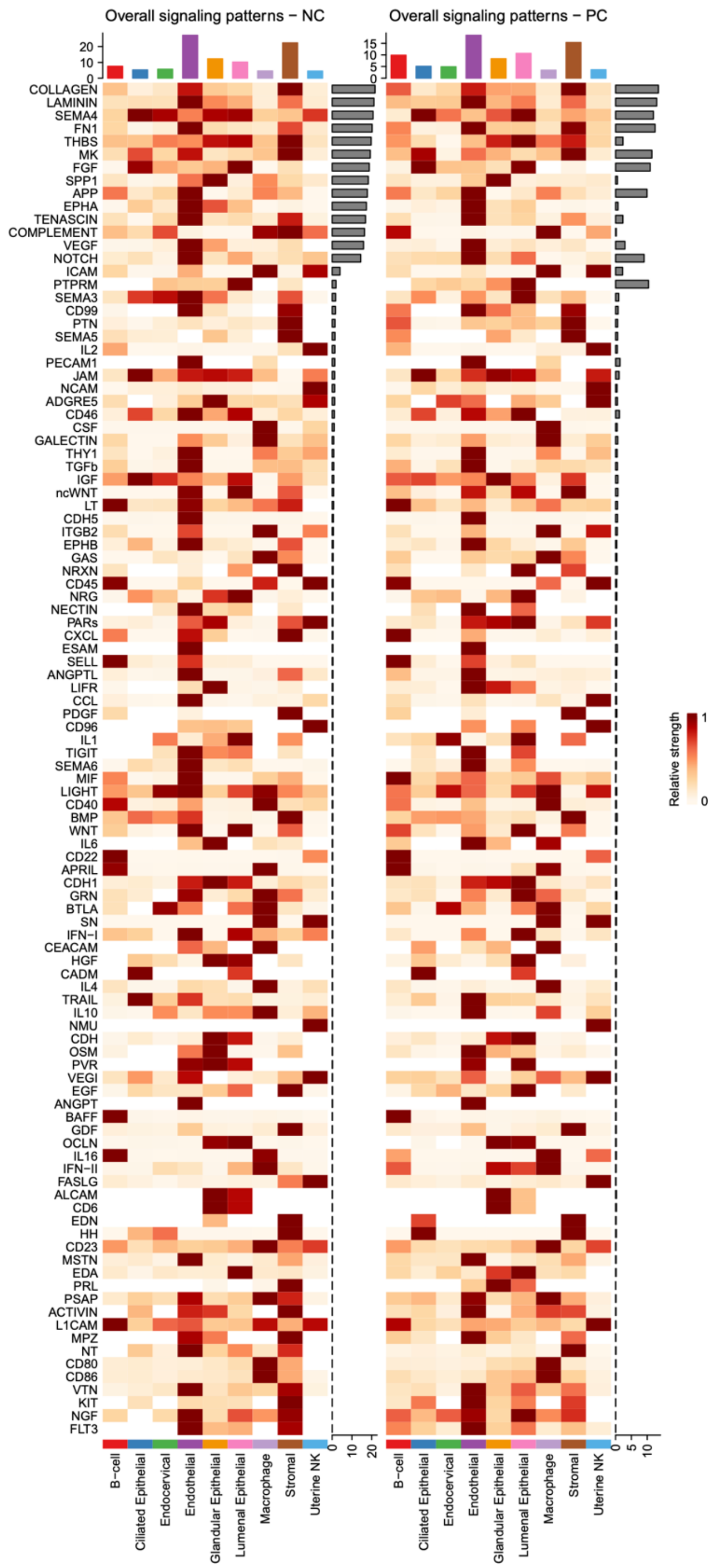

Supplemental Figure S4. Comparative heatmap of overall signaling patterns in NC versus PC WOI endometrium by cell type, demonstrating signaling intensity differences in various pathways

Supplemental Table S1. Relevant patient data of endometrium samples for single-nucleus sequencing experiment

	NC endometrium (n=9)	PC endometrium (n=7)	p-value
Age (median, IQR)	36 (35 – 38)	38 (35 – 45)	0.31
Body Mass Index (median, IQR)	21.8 (21.0 – 26.2)	23 (21.6 – 23.2)	0.92
Primary infertility diagnosis	Unexplained infertility	Unexplained infertility	n/a
Nulliparity (%)	88.9%	71.4%	0.38
Uterine/endometrial abnormality	None	None	n/a
Biopsy timing	LH+7	6th day of PIO	n/a
Biopsy method	Endometrial Pipelle	Endometrial Pipelle	n/a
Endometrial thickness, mm (median, IQR)	Not measured per clinic protocol	9.1 (8.3 – 10.2)	n/a
Prior miscarriage (median, IQR)	0 (0 – 2)	0 (0 – 1)	0.56
Prior failed euploid embryo transfer attempts (mean, min - max)	0.71 (0 – 2)	0.67 (0 – 3)	0.92

*p-value by Mann-Whitney U test or χ^2 test as appropriate

Supplemental Table S2. Hormonal medication regimen in programmed cycle patients

Sample #	Estrogen exposure	Progesterone exposure
1	Estradiol patch 300mcg every 3 days, totaling 16 days prior to progesterone exposure	Progesterone-in-oil intramuscular injection 50mg daily
2	Estradiol patch 300mcg every 3 days, totaling 13 days prior to progesterone exposure	Progesterone-in-oil intramuscular injection 50mg daily
3	Estradiol patch 300mcg every 3 days, totaling 20 days prior to progesterone exposure	Progesterone-in-oil intramuscular injection 50mg daily
4	Estradiol patch 300mcg every 3 days, totaling 13 days prior to progesterone exposure	Progesterone-in-oil intramuscular injection 50mg daily
5	Estradiol patch 300mcg every 3 days, totaling 16 days prior to progesterone exposure	Progesterone-in-oil intramuscular injection 50mg daily
6	Estrace tablet 6mg daily, totaling 16 days prior to progesterone exposure	Progesterone-in-oil intramuscular injection 50mg daily
7	Estradiol patch 300mcg every 3 days, totaling 13 days prior to progesterone exposure	Progesterone-in-oil intramuscular injection 50mg daily

Supplemental Table S3. Baseline clinical characteristics of clinical cohort

	**Participants, No (%)**			
	**Non-ART + NC (n = 208)**	**ART-embryo +NC (n = 83)**	**ART-embryo +PC (n = 257)**	**p-value**
**Maternal Age, median (IQR), y**	36 (34-38)	38 (35-40)	39 (36-43)	<0.01
**Paternal Age, median (IQR), y**	36 (34-40)	39 (36-42)	39 (36-44)	<0.01
**BMI, median (IQR)**	23.9 (21.6-26.6)	23.2 (21.3-25.6)	23.9 (21.4-26.9)	0.33
**Primary infertility diagnosis**				
Unexplained	79 (38.0)	34 (41.0)	105 (40.9)	<0.01
PCOS	37 (17.8)	0 (0)	14 (5.4)	
Other ovulatory	21 (10.1)	2 (2.4)	7 (2.7)	
Male Factor	19 (9.1)	19 (22.9)	38 (14.8)	
Tubal	0 (0)	7 (8.4)	11 (4.3)	
Severe endometriosis	1 (0.5)	2 (2.4)	11 (4.3)	
Asherman's	7 (3.4)	0 (0)	4 (1.5)	
Combined male/female	1 (0.5)	5 (6.0)	39 (15.2)	
Recurrent pregnancy loss	25 (12.0)	5 (6.0)	15 (5.8)	
Other	18 (8.6)	9 (10.9)	13 (5.1)	
**Pre-pregnancy hypertension**				
No	202 (97.1)	83 (100)	246 (95.7)	0.13
Yes	6 (2.9)	0 (0)	11 (4.3)	
**Pre-pregnancy diabetes**				
No	205 (98.6)	83 (100)	253 (98.4)	0.77
Yes	3 (1.4)	0 (0)	4 (1.6)	
**Previous livebirth**				
No	148 (71.2)	46 (55.4)	150 (58.4)	<0.01
Yes	60 (28.8)	37 (44.6)	107 (41.6)	
**Prior miscarriage history**				
None	139 (66.8)	54 (65.1)	178 (69.3)	0.73
One miscarriage	40 (19.2)	19 (22.9)	53 (20.6)	
Two or more miscarriages	29 (14.0)	10 (12.0)	26 (10.1)	
**Maternal Race/Ethnicity**				
White/Caucasian	143 (68.8)	48 (57.8)	157 (61.3)	0.36
Asian/Pacific Islander	55 (26.4)	30 (36.2)	81 (31.6)	
Hispanic/Latino	3 (1.4)	3 (3.6)	8 (3.1)	
Black/African American	1 (0.5)	0 (0)	5 (2.0)	
Multiracial	6 (2.9)	2 (2.4)	5 (2.0)	

*p-value by Kruskal-Wallis test, χ^2 test, or Fisher's exact test as appropriate

Supplemental Table S4. Risk ratios of HDP diagnoses by mode of conception

	n/N (%)	Crude relative risk		Adjusted relative risk[a]	
**HDP[b]**		**RR (95% CI)**	**p-value**	**aRR (95% CI)**	**p-value**
Mode of conception					
(ref: non-ART+NC)	43/208 (20.7%)	ref		ref	
ART+NC	14/83 (16.9%)	0.816 (0.472–1.410)	0.466	0.831 (0.398–1.738)	0.624
ART+PC	53/257 (20.6%)	0.998 (0.697–1.427)	0.989	0.905 (0.530 –1.548)	0.716
**Preeclampsia[b]**					
Mode of conception					
(ref: non-ART+NC)	26/208 (12.5%)	ref		ref	
ART+NC	9/83 (10.8%)	0.867 (0.425–1.772)	0.696	1.035 (0.424–2.528)	0.939
ART+PC	34/257 (13.2%)	1.058 (0.657–1.704)	0.816	0.981 (0.512–1.878)	0.954
**Preeclampsia with severe features[b]**					
Mode of conception					
(ref: non-ART+NC)	16/208 (7.7%)	ref		ref	
ART+NC	6/83 (7.2%)	0.940 (0.381–2.319)	0.893	0.992 (0.337–2.927)	0.989
ART+PC	23/257 (8.9%)	1.163 (0.631–2.144)	0.627	0.994 (0.452–2.185)	0.987
**Preterm preeclampsia with severe features[c]**					
Mode of conception					
(ref: non-ART+NC)	12/208 (5.8%)	ref		ref	
ART+NC	3/83 (3.6%)	0.627 (0.181–2.163)	0.460	0.696 (0.174–2.781)	0.608
ART+PC	15/257 (5.8%)	1.012 (0.484–2.113)	0.975	0.740 (0.291–1.886)	0.529

[a]adjusted for maternal age, BMI, nulliparity, infertility diagnosis, and aspirin use; p-value by univariable or multivariable log-binomial regression as appropriate

[b]Hypertensive disorders of pregnancy, including gestational hypertension, preeclampsia, and preeclampsia with severe features were defined using the criteria set forth by the American College of Obstetricians and Gynecologists Practice Bulletin #222: Gestational Hypertension and Preeclampsia

[c]Preterm preeclampsia was defined as diagnosis at <37 weeks gestational age

Supplemental Table S5. Additional placental-mediated obstetric complications by mode of conception

	Participants, No (%)			
	**Non-ART + NC (n = 208)**	**ART-embryo +NC (n = 83)**	**ART-embryo +PC (n = 257)**	**p-value[b]**
**Placental abruption[a]**				
**Yes**	2 (0.96)	0 (0.00)	7 (2.72)	0.21
**No**	206 (99.05)	83 (100.00)	250 (97.28)	
**Placenta previa**				
**Yes**	2 (0.96)	0 (0.00)	2 (0.78)	0.68
**No**	206 (99.05)	83 (100.00)	255 (99.22)	
**Fetal growth restriction**				
**Yes**	5 (2.40)	1 (1.20)	5 (1.95)	0.85
**No**	203 (97.60)	82 (98.80)	252 (98.05)	
**Gestational diabetes**				
**Yes**				
Diet-controlled	13 (6.25)	4 (4.82)	10 (3.89)	0.14
Hypoglycemic agent	14 (6.73)	10 (12.05)	12 (4.67)	
**No**	181 (87.02)	69 (83.13)	235 (91.44)	

[a]Clinically significant placental abruption necessitating admission and/or delivery
[b]p-value by Fisher's exact test
